# CXCR4 expression heterogeneity in neuroblastoma cells due to ligand-independent regulation

**DOI:** 10.1186/1476-4598-8-126

**Published:** 2009-12-22

**Authors:** Alex J Carlisle, Christopher A Lyttle, Rosalind Y Carlisle, John M Maris

**Affiliations:** 1 Division of Oncology, Abramson Research Center, Children's Hospital of Philadelphia, ARC-907A, 3615 Civic Center Blvd., Philadelphia, Pennsylvania 19104-4399, USA

## Abstract

**Background:**

CXCR4, the receptor for the chemokine stromal-derived factor 1 (SDF-1), has been shown to mediate many of the processes essential for cancer progression such as tumor cell proliferation, metastasis, and angiogenesis. To understand the role of CXCR4 in the biology of neuroblastoma, a disease that presents with wide spread metastases in over 50% of patients, we screened ten patient derived-neuroblastoma cell-lines for basal CXCR4 expression and sought to identify characteristics that correlate with tumor cell phenotype.

**Results:**

All cell lines expressed *CXCR4 *mRNA at variable levels, that correlated well with three distinct classes of CXCR4 surface expression (low, moderate, or high) as defined by flow cytometry. Analysis of the kinetics of CXCR4 surface expression on moderate and high expressing cell lines showed a time-dependent down-regulation of the receptor that directly correlated with cell confluency, and was independent of SDF1. Cell lysates showed the presence of multiple CXCR4 isoforms with three major species of approximately 87, 67 and 55 kDa associating with high surface expression, and two distinct species of 45 and 38 kDa correlating with low to null surface expression. Western blot analysis of CXCR4 immunoprecipitates showed that the 87 and 67 kDa forms were ubiquitinated, while the others were not. Finally, treatment of cells with a proteasome inhibitor resulted in down regulation of CXCR4 surface expression.

**Conclusions:**

Taken together, these data show that regulation of CXCR4 surface expression in neuroblastoma cells can occur independently of SDF-1 contribution arguing against an autocrine mechanism. Additionally these data suggest that post-translational modifications of CXCR4, in part through direct ubiquitination, can influence trafficking of CXCR4 to the surface of neuroblastoma cells in a ligand-independent manner.

## Background

Neuroblastoma, a pediatric cancer of the sympathetic nervous system, accounts for roughly 15% of cancer related deaths in children [1]. A variety of clinical and biological parameters are currently used for risk-assessment and outcome prediction, but morbidity and mortality remain unacceptable, and an urgent need for improved therapies exists [2]. One emerging candidate for targeted therapy in cancer and several other diseases is CXCR4 [3-6]. CXCR4 has been shown to be involved in the major aspects of cancer progression, tumor cell proliferation, metastasis, and angiogenesis, and its involvement in one or more of these processes has been demonstrated in several human malignancies [7-9]. CXCR4, a seven transmembrane domain receptor expressed on the surface of a variety of cells, serves as the receptor for the chemokine SDF-1, also known as CXCL12. Through interaction with its ligand, CXCR4 mediates one of the signals that influence migration of various cells to their endpoint destinations. The most direct evidence for the biological significance of CXCR4 is seen in murine knockout models which result in lethality via impaired development of their hematopoietic, circulatory, and nervous systems [10,11], indicating an essential role for SDF-1/CXCR4 signaling in normal development. In humans, genetic mutation of *CXCR4 *is associated with the clinical syndrome WHIM, in which a truncating mutation results in deregulation of receptor signaling and consequent impairment of leukocyte function [12,13]. The biological importance of CXCR4 is further underscored by the observation that disruption of the SDF-1/CXCR4 axis has consequence on essential physiological processes such as homing of hematopoietic stem cells to marrow [14] and migration of immune cells to inflammatory sites [15], as well as patho-physiological processes such as HIV uptake by host cells [16] and cancer progression. Despite its obvious importance in the progression of several forms of cancer, the role and nature of CXCR4 function in neuroblastoma biology is less clear. CXCR4 has been shown to mediate proliferation of neuroblastoma cells under certain conditions [17]. Additionally, despite reporting little to no basal surface expression of CXCR4 on the majority of neuroblastoma cells they examined one group has reported SDF-1 mediated chemotaxis and down-regulation of CXCR4 upon derivatizing one of their cell lines to overexpress CXCR4 [18]. Others have suggested however that CXCR4 is not involved in chemotaxis of neuroblastoma cells [19] and that SDF-1/CXCR4 signaling causes cell death [20]. In light of these findings the extent to which basal CXCR4 surface expression occurs in neuroblastoma cells and the significance in of this expression on the biology of these tumors has not been conclusively demonstrated. Previous work in our lab employing a transcriptome wide analysis of a large neuroblastoma sample set was performed to identify genes associated with the disease and to further refine its classification [21]. Analysis of results from this data set identified *CXCR4 *as one of the genes differentially overexpressed in metastatic compared to localized primary tumors obtained at diagnosis, supporting a role for CXCR4 in neuroblastoma dissemination. In an effort to further assess CXCR4 expression patterns in neuroblastoma and the correlation of such patterns with tumor progression, global expression of CXCR4 in neuroblastoma cells was analyzed at the transcriptional, translational, and surface expression level.

## Materials and methods

### Cell Culture

Ten human neuroblastoma cell lines (CHP-134, KCN, KCNR, LAN-5, NB-69, NGP, SH-SY5Y, SK-N-AS, SK-N-FI, SK-N-SH), all derived from the tumors of patients with advanced stage neuroblastoma (Stage 3 or 4) were obtained from the Children's Oncology Group (COG) and cultured in 75 cm^2 ^cell culture flasks (corning) using RPMI-1640, supplemented with 10% fetal bovine serum, 2 mM glutamine, and a 1/100 dilution of penicillin/streptomycin/amphotericin B. Cells were grown at 37°C in a humidified environment of 95% air and 5% CO_2 _(All cell culture media reagents were from Invitrogen). SK-N-ASΔ3 was generated by subcloning an expression vector containing the coding sequence for the E3 ubiquitin ligase cullin-5. Briefly, full length double stranded Cul-5 cDNA was amplified from total SK-N-AS RNA with primers designed using Primer3 software http://frodo.wi.mit.edu/primer3/. Cul-5 cDNA was gel-purified by band excision and extraction using a QIAquick gel extraction kit (Qiagen) followed by sequencing to confirm transcript identity. Purified Cul-5 cDNA was sub-cloned into a pcDNA4 expression vector using a pcDNA4/HisMax TOPO^® ^TA Expression Kit (Invitrogen) and competent E-coli transformed following manufacturer's protocol. Positive transformants were identified using ampicillin selective media followed by sequence analysis to confirm orientation. After generation, Cul-5 expression vectors were transfected into SK-N-AS cells using Lipofectamine 2000 (Invitrogen) and stable transfectants selected using zeocin. Unless otherwise indicated all cells were grown to 80-90% confluency.

### Fluorescence-activated Cell Sorting (FACS) Assay

Neuroblastoma cells were cultured as described previously. For Brefeldin A studies cells were cultured in the presence of 1 μg/ml Brefeldin A, or a corresponding volume of DMSO for 7 hrs, prior to harvesting. For Lactacystin studies cells were cultured in the presence of 10 μM Lactacystin or corresponding volume of PBS overnight. After culturing, cells were harvested using Versene (0.02% EDTA in hanks balanced salt solution) and washed twice, once with culture media, and once with cold PBA (Phosphate buffered saline containing 2%BSA and 0.1% NaN_3_, pH 7.4). Once washed, cells were adjusted to a concentration of 1 × 107 cells per ml in PBA and 50 μl of cells were incubated with 20 μl of the PE-conjugated mouse anti-human CXCR4 antibody 12G5 (BD Biosciences) at room temperature for 30 minutes in the dark. Following incubation with antibody, cells were washed three times in PBA and analyzed on a BD FACSCaliber Flow Cytometer using CellQuest Pro Software (BD Biosciences). For negative controls PE-conjugated mouse IgG_2a _was used. One thousand events were counted for each sample to generate a distribution of fluorescent intensities. The geometric mean of those intensities was normalized to the geometric mean obtained from corresponding mouse IgG_2a _stained cells. Final data is represented as relative mean fluorescent intensity from three experiments.

### Quantitative PCR

Cells were cultured and harvested as previously described. Total RNA was isolated from 1 × 10^7 ^cells using the Absolutely RNA miniprep kit (Stratagene) following manufacturers instructions followed by 1^st ^strand cDNA synthesis from 5 μg total RNA using the SuperScript Firs-Strand Synthesis System for RT-PCR (Invitrogen) following manufacturers instructions. Quantitative PCR was performed using the CXCR4 TaqMan Gene Expression Assay Hs00607978 along with simultaneous amplification of glyceraldehyde phosphate dehydrogenase (GAPDH) as an endogenous control. Thirty cycles of amplification were performed and the difference between CXCR4 and GAPDH cycle threshold values (dCT) were graphed as 1/dCT. Detection of cul-5 transcripts was performed with the same method with exception of using TaqMan Gene Expression Assay Hs00967483_m1. Assays were done in triplicate

### SDF-1 Enzyme-Linked ImmunoSorbent Assay (ELISA)

Cells were cultured as previously described. Prior to harvesting, conditioned media was collected and concentrated from initial volume of 12 ml to 500 μl by centrifuging in a Centriplus Model YM-3 centrifugal filter device at 3000 × g and 4°C in a JA-20 rotor. After concentration, 100 μl aliquots of conditioned media from cell lines were placed into 96 well microplates coated with murine anti-human SDF-1α monoclonal antibody and ELISA based detection of SDF-1 was performed using the Quantikine Human SDF-1α Immunoassay kit (R&D Systems). Absolute quantification of SDF-1 levels were determined by interpolating from a curve of standard SDF-1 concentrations included on the assay plate; unconditioned culture media was used to correct for background levels of SDF-1 in the unconditioned culture media. Assays were performed in triplicate.

### Immunofluorescent labeling of CXCR4 and SDF-1 in Neuroblastoma Cells

Sterile 15 mm glass coverslips (Fisher) were placed into 150 mm culture dishes containing supplemented RPMI-1640. SK-N-SH or SH-SY5Y cells were seeded onto coverslips and grown at 37°C in a humidified environment of 95% air and 5% CO_2_. At 80% confluency cells were rinsed twice with PBS gently in culture dish so as not to become detached, briefly fixed with a 3.7% formaldehyde solution in PBS, and washed twice more with PBS. Fixed Cells were permeabilized using a 10% NP-40 solution in PBS followed by washing once in PBS and then transfer to a new culture dish containing moist paper towels to prevent coverslips from drying. Coverslips were blocked with PBG, a modified PBS solution containing 0.2% fish gelatin (sigma) and 0.5% BSA (sigma), for a minimum of 30 minutes. Blocked coverslips were then incubated with 1/1000 dilutions of mouse anti-CXCR4 monoclonal antibody MAB172 and goat anti-SDF-1 antibody MAB310 (R&D Systems) for either 1 hr at room temperature or overnight at 4°C. After incubation with primary antibodies coverslips were washed three times with PBG followed by incubation with 1/200 dilutions of FITC-conjugated donkey anti-mouse and TRITC-conjugated donkey anti-goat secondary antibodies (Jackson ImmunoResearch) for 1 hr at room temperature or 4°C overnight. Following incubation with secondary antibodies coverslips were washed three times with PBG with the inclusion of 0.5 μg/ml of 4', 6-diamino-2-phenylindole (Sigma) in the third wash. Coverslips were then washed twice with PBS, mounted onto 3'' × 1'' × 1 mm glass microscope slides (Fisher) with Gel/Mount embedding medium containing anti-fading agents (Biomedia), and sealed with liquid enamel (Revlon). Fluorescently labeled cells were visualized using immune complexes were visualized using an inverted fluorescent microscope. Figure shows a superimposed composite of CXCR4 and SDF-1 imaging.

### Western Blot Detection of CXCR4

Cells were cultured as previously described. After harvesting 1 × 10^7 ^cells were washed twice in PBS and solubilized in 1 ml of lysis buffer consisting of 50 mM Tris base, 150 mM NaCl, 1% Triton X-100 pH 7.5. Cell lysates were centrifuged at 14, 000 rpm and 4°C for 30 minutes in a table-top refrigerated centrifuge (Eppendorf) and supernatants were analyzed for total protein content by performing the DC Protein Assay (Bio-Rad) and measuring absorbance at 750 nm on a Benchmark Plus Microplate Spectrophotometer (Bio-Rad). Total protein concentrations in cell line samples were determined by interpolation from a linear curve of BSA values. Proteins were resolved by electrophoresis using the Xcell SureLock Mini-Cell system (Invitrogen) following manufacturer's instructions. Briefly, 1 μg of total protein for each cell line was loaded onto a NuPAGE 4-12% Bis-Tris Gel followed by electrophoresis at 200 constant volts for 40 minutes. High-Range Rainbow Molecular Weight Markers (Amersham Biosciences) were simultaneously run with samples in separate lanes to monitor resolution. Following electrophoresis resolved proteins were transferred to Hybond ECL Nitrocellulose membranes (Amersham Biosciences) at 30 constant volts for 1 hr at room temperature using an Xcell II Blot Module (Invitrogen) following manufacturer's instructions. Transfer efficiency was assessed by briefly staining membranes with a 0.1% Ponceau S solution in 5% Acetic acid (Sigma) followed by rinsing in deionized distilled H2O. After removal of Ponceau S western blot detection was performed using an ECL Western Blot Detection Kit (Amersham Biosciences) following manufacturer's instructions. Briefly, membranes were blocked in 5% blocking solution, rinsed in TBS pH 7.6 containing 0.1% Tween 20 (TBS-T), and incubated with a 1/1000 dilution of the rabbit anti-human CXCR4 antibody ab2090 (abcam) at either room temperature for 1 hr or overnight at 4°C. Following adsorption of primary antibody, membranes were rinsed in TBS-T and then incubated with a 1/2000 dilution of horseradish peroxidase (HRP) conjugated anti-rabbit antibody for 1 hr at room temperature. Following incubation with secondary antibody membranes were washed extensively and a chemiluminescent substrate for 1 min at room temperature. Labeled molecules were visualized by 30 second dark room exposure onto Hyperfilm ECL chemiluminescence film in a BioMax Cassette (Kodak) and development on an M35 X-OMAT processor (Kodak). Determination of molecular mass was performed by linear interpolation from a curve of chemiluminescent standards (Amersham Biosciences) run along side samples during electrophoresis. For Cul-5 western blot detection procedure was the same as for CXCR4 detection with the exception of using a 1/200 dilution of ab82292, a rabbit polyclonal antibody specific for the carboxy terminus of CUL-5 (abcam), as the primary antibody. For Ubiquitin western blot detection lysates were adsorbed with 10 μg/ml of the rabbit anti-human antibody OPA1-01100 (Affinity BioReagents) overnight at 4°C. Immune complexes were bound to pre-washed Protein G Agarose beads (Invitrogen) by incubating for 1 hr at room temperature followed by precipitation by centrifugation at 10, 0000 × g for 10 minutes at room temperature. CXCR4 immunoprecipitates were washed extensively with lysis buffer and TBS-T prior processing in sample buffer and loading onto gels for electrophoresis. Western blot detection of ubiquitin in CXCR4 immunoprecipitates was performed similarly as for CXCR4 western blots except membranes were incubated with the mouse anti-human ubiquitin primary antibody MAB701 (R&D Systems) and an HRP-conjugated anti-mouse secondary antibody (Amersham Biosciences) at 1/1000 and 1/2000 respectively.

## Results

### CXCR4 is Differentially Expressed in Neuroblastoma Cells

**Figure 1:**
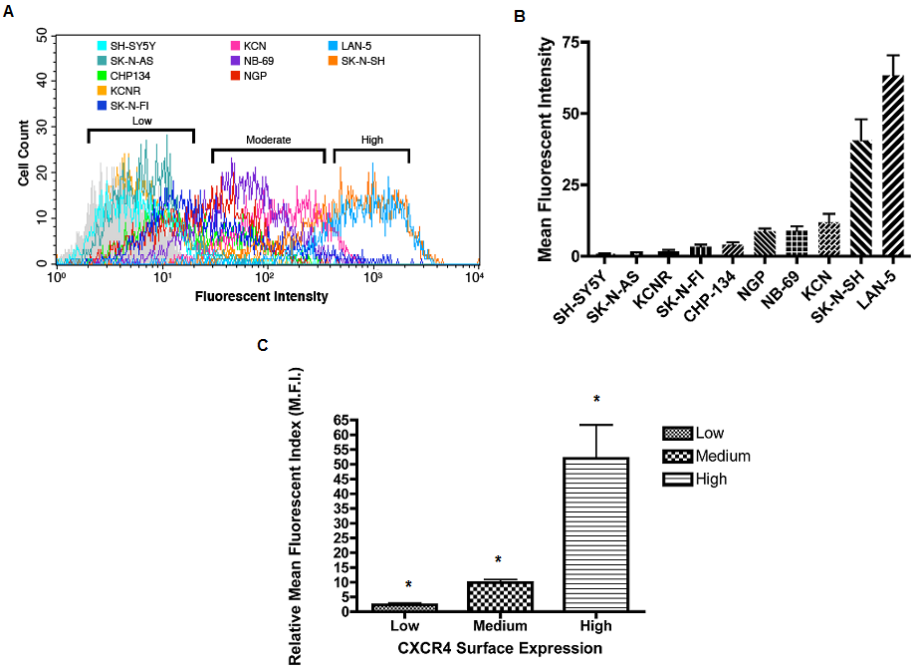
**CXCR4 is Differentially Expressed on the Surface of Neuroblastoma Cells**. Neuroblastoma cells were cultured to 80% confluency, harvested with versene followed by washing in culture media, then a modified phosphate buffered saline (PBA). 5 × 10^5 ^intact and non-permeabilized cells were incubated with a PE-conjugated Anti-CXCR4 monoclonal antibody (12G5) for 30 minutes, washed and flow cytometry was performed to measure cell surface associated fluorescence. **A) **Frequency histogram showing distribution of fluorescent intensities associated with cell surface of labeled cells. Fluorescent intensity is shown on the x-ordinate and the number of cell events on the y-ordinate. A total of 1000 events were recorded for each measurement. Three distinct ranges of distribution are evident (brackets). Data shown is from one experiment and is representative of results obtained from three experiments. **B) **The geometric mean fluorescent intensity for each cell line was determined after staining with anti-CXCR4 antibody, this value was then normalized to the corresponding geometric mean fluorescent intensity obtained from surface staining each cell line with the PE-conjugated isotype matched control antibody (Mouse IgG2a). Final quantitative values are represented as a relative mean fluorescent intensity (M.F.I.) +/- SD obtained, from three experiments. Means were stratified into three classes of surface expression and the following cutoffs were used to define each class: Low= M.F.I. <5; Medium = M.F.I. 5-20; High = M.F.I. >20 (see table 1). **C) **The mean plus SD was determined for each surface expressing class using the intensities of all the cell lines in that class. The mean value of each class falls within the parameters used to define that class. All means were significantly different from one another. (*P < 0.0001, One-way ANOVA).

**Figure 2:**
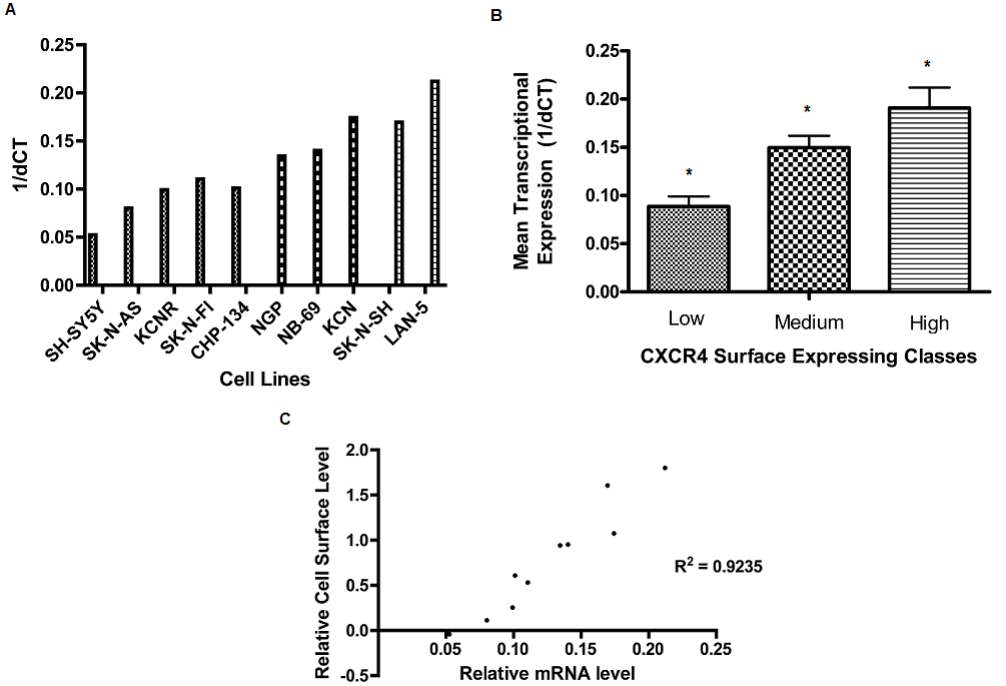
**Transcriptional Expression of CXCR4 in Neuroblastoma Cells**. Total RNA was isolated from each cell line and used to generate cDNA templates. Quantitative PCR was performed using a total of 250 ng of template cDNA for each cell line and 30 cycles of amplification. **A) **CXCR4 mRNA levels in panel of ten neuroblastoma cell lines. dCt values for CXCR4 were normalized to the endogenous control glyceraldehyde phosphate dehydrogenase. dCt values represent the average of three replicates. **B) **The mean dCt value for each surface expression class was generated from all dCt values of cell lines in a given class. Result shows the mean dCt +/- SD of each class to be significantly different from one another (P < 0.005, One-way ANOVA). **C) **The log_10 _of mean fluorescent intensity was plotted against the mean dCt for each cell line. Each data point represents the mean value obtained from three FACS assays plotted against the mean value obtained from three quantitative PCR assays. Figure shows a strong and significant correlation between the two forms of expression (Pearson correlation, R^2 ^= 0.9235, P < 0.05).

Ten human neuroblastoma-derived cell lines from patients with high-risk disease were screened for surface expression of CXCR4 by flow cytometry. An overlay of the representative frequency distribution of fluorescence intensities for each cell line resulted in the peak intensities clustering into one of three distinct regions (Fig. 1A). Fig 1B shows a quantitative representation of the range of normalized geometric mean intensities from three experiments for each of the cell lines. The M.F.I. for each cell line was assigned to one of three classes of expression based on the following cutoffs; <5 for low, 5-20 for medium, and greater than 20 for high. Fig. 1C shows a comparison of the mean value of intensities in the three defined classes to demonstrate the significant difference between them as determined by one-way ANOVA analysis (P < 0.0001). Table 1 shows the assignment of each cell line to one of the three defined surface expression classes. To determine if the observed variability in CXCR4 surface expression was related to transcriptional regulation, quantitative RT-PCR was performed to compare CXCR4 mRNA copy number in the panel of neuroblastoma cell lines. Fig. 2A shows a similar pattern of heterogeneity with regard to CXCR4 transcriptional expression. With the exception of three cell lines, CHP-134, NB-69, and SK-N-SH, the same order of expression seen at the surface level was maintained at the transcriptional level. The same class assignment given in surface expression analysis was used for assessing transcriptional expression and as can be seen in fig. 2B this similarly resulted in the generation of three statistically distinct classes of mean transcriptional expression (P < 0.005, one-way ANOVA). To determine how closely transcriptional expression reflects the expression of CXCR4 at the cell surface, nonlinear regression analysis was performed using the respective means for each cell line. Fig. 2C shows there was a strong correlation (Pearson correlation, R^2 ^= 0.9235, P < 0.05) between the two types of expression but that transcription alone was not a complete indication of CXCR4 surface levels.

**Table 1:** Classification of Neuroblastoma Cell Lines by CXCR4 Surface Expression

CELL LINE	MEAN FLUORESCENT INTENSITY	CXCR4 SURFACE EXPRESSION CLASS
SH-SY5Y	0.9086	Low

SK-N-AS	1.290	Low

KCNR	1.796	Low

SK-N-FI	3.569	Low

CHP-134	4.066	Low

NGP	8.772	Moderate

NB-69	8.99	Moderate

KCN	11.899	Moderate

SK-N-SH	40.57	High

LAN-5	63.33	High

### Autocrine Mediated Down-Regulation of CXCR4 does not occur in Neuroblastoma Cells

**Figure 3:**
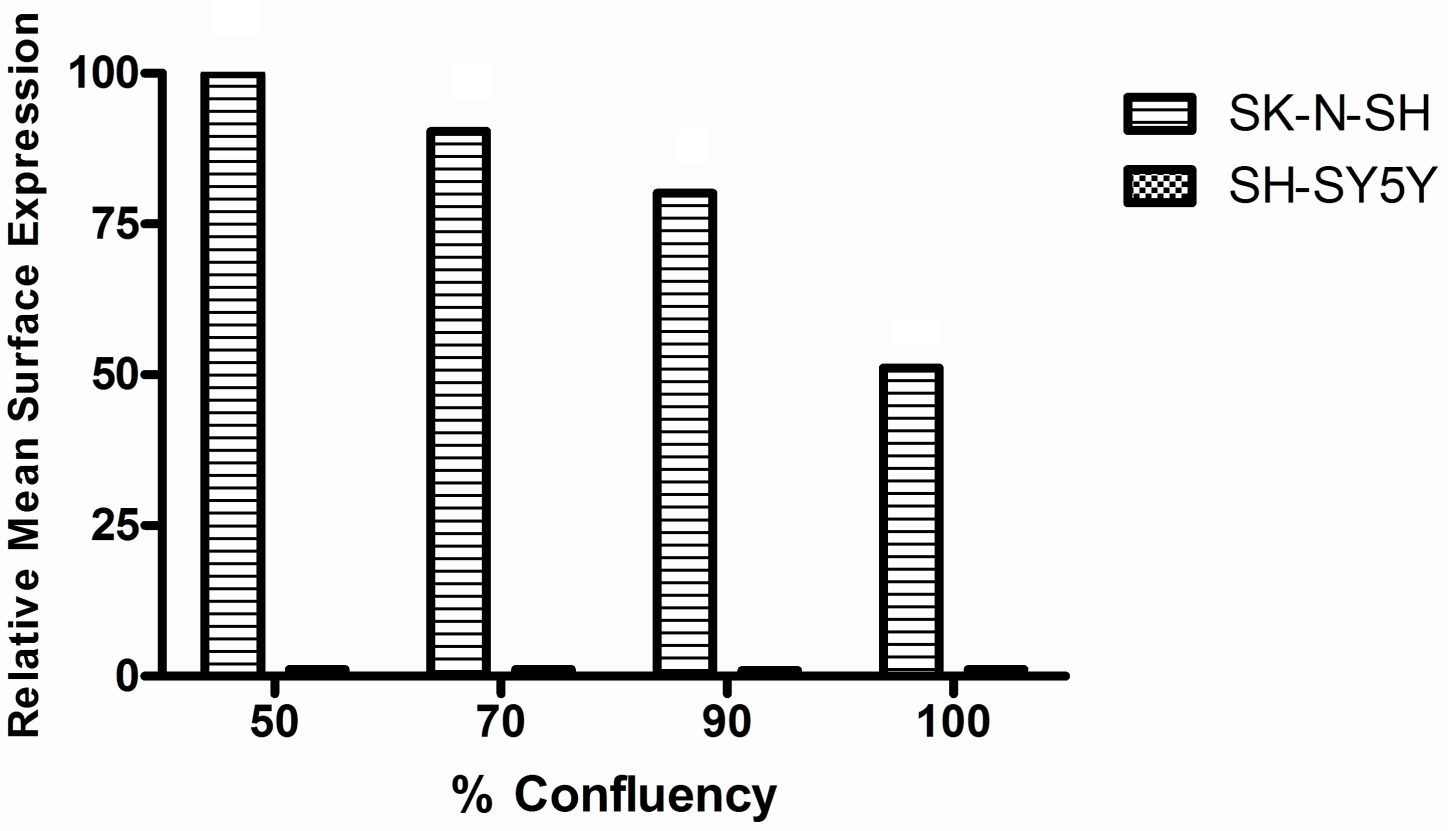
**Effect of Cell Density on CXCR4 Surface Expression**. Individual flasks of culture media were each synchronously seeded with 5 × 10^6 ^SK-N-SH cells and grown at 37°C to varying confluency. At each confluency, cells were harvested and processed for cell surface staining of CXCR4. The maximum CXCR4 signal was observed at 50% confluency and set as 100% mean fluorescent intensity. All intensities are represented as % relative values normalized to the maximum intensity. Result represents the mean from three experiments and shows the correlation between cell density and surface expression (Pearson correlation, R^2 ^= 0.9488, P < 0.05).

Previous reports have shown that CXCR4 is down-regulated at the neuroblastoma cell surface in response to exogenously added SDF-1 leading to the hypothesis that CXCR4 surface expression is regulated by a negative autocrine feed back loop mediated by an accumulation of SDF-1 secreted from neuroblastoma cells [18]. In an effort to assess the possible effects of endogenously derived SDF-1 accumulated during cell culture on CXCR4 surface expression, cells at different degrees of confluency were measured for CXCR4 at the cell surface using flow cytometry. As shown in fig. 3, increasing confluency resulted in a progressive decrease in CXCR4 at the surface of cells in the higher surface expressing classes, demonstrating a significant correlation between cell density and surface expression of the receptor (Pearson correlation analysis, R2 = 0.9488; P < 0.05); this observation was consistent for all of the moderate to high CXCR4 surface expressing cell lines tested. SH-SY5Y, a sub-clone of SK-N-SH and a low surface expressing cell line showed no difference in CXCR4 surface expression at any confluency; this observation was consistent for all cells in the low CXCR4 surface expressing class. Maximal CXCR4 surface expression was observed for cell cultures less than 50% confluent (data not shown), and upon 100% confluency CXCR4 surface expression was reduced to half maximal levels.

**Figure 4:**
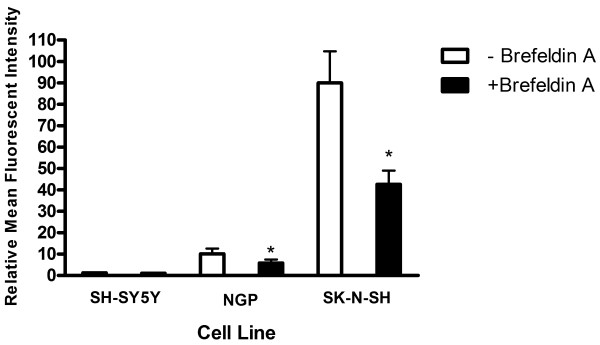
**Effect of the Secretion Inhibitor Brefeldin A on CXCR4 Surface Expression**. Various cell lines from all three surface expression classes were cultured with the addition of 1 μg/ml Brefeldin A. Cells were left at 37°C for 12 hrs, harvested, washed, and processed for FACS analysis. Result represents the M.F.I. +/- SD from three experiments (*P < 0.05, Paired t-test).

**Figure 5:**
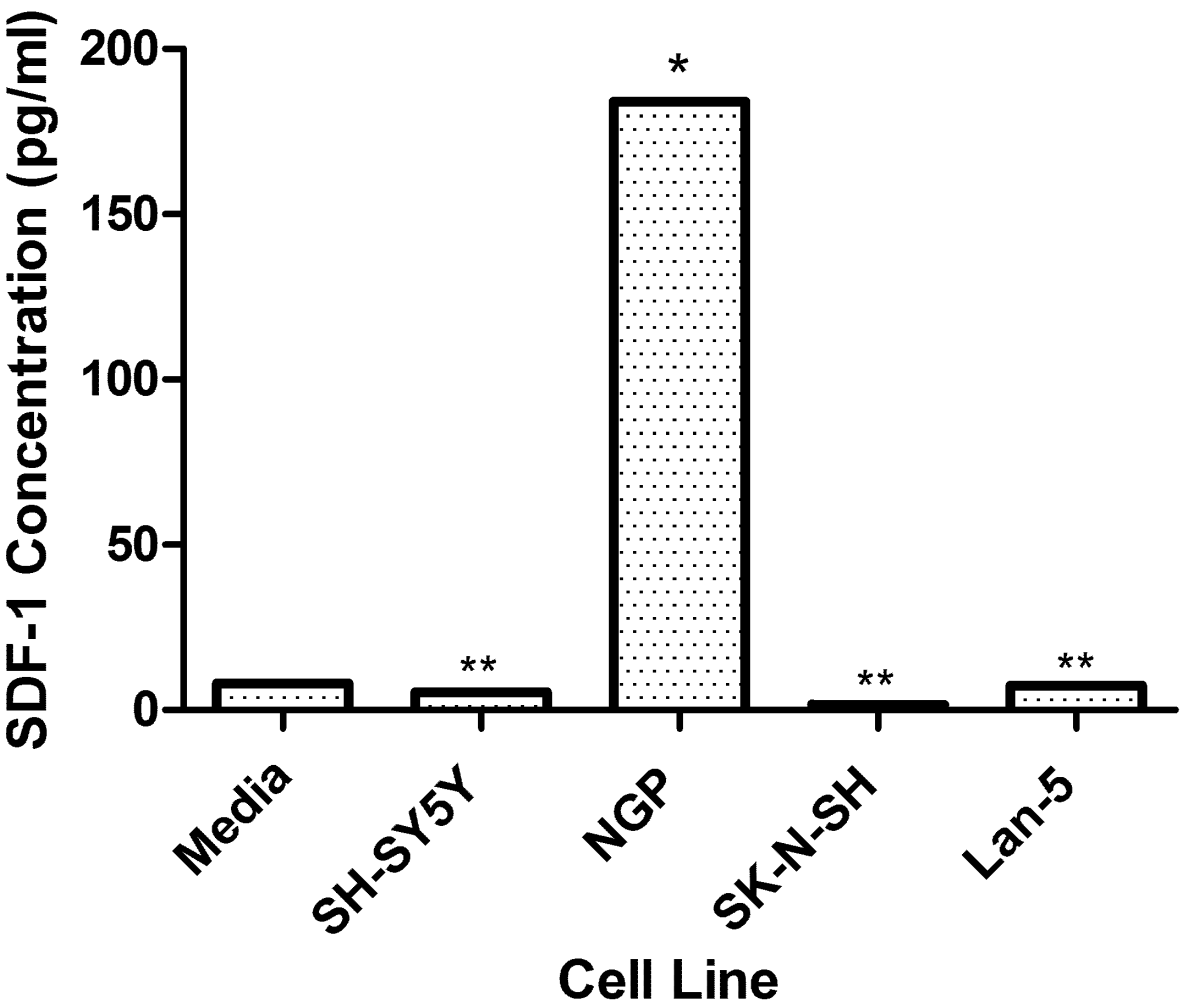
**Quantitative Analysis of SDF-1 Secretion by Neuroblastoma Cells**. Conditioned media from several neuroblastoma cell lines was collected at maximum confluency. Conditioned media was concentrated from 12 ml to 0.5 ml by filter centrifugation using membranes with 3000 molecular weight cut-offs. Quantitative Analysis of SDF-1 was performed using an enzyme linked immunoadsorbent assay (ELISA). Result represents the mean of three experiments for representatives from each surface expressing class compared to media background levels (*P < 0.005 and **P > 0.05, unpaired t-test).Concentrations are expressed in pg/ml.

**Figure 6:**
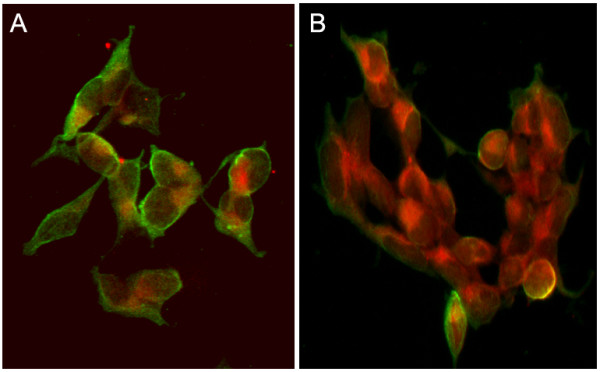
**Immunofluorescent Staining of CXCR4 and SDF-1 in Neuroblastoma Cells**. Cells were seeded onto coverslips and grown to 80% confluency. At desired confluency cells were fixed with 3.7% formaldehyde, permeabilized with 10% NP-40 and blocked with PBG. CXCR4 and SDF-1 immune complexes were generated by incubating cells with 1/1000 dilutions of the mouse anti-CXCR4 monoclonal antibody MAB172 and the goat anti-SDF-1 antibody MAB310 respectively. 1/200 dilutions of FITC-conjugated donkey anti-mouse and TRITC-conjugated donkey anti-goat antibodies were used to localize CXCR4-MAB172 complexes respectively. Immune complexes were visualized using an inverted fluorescent microscope. Figures show superimposed composites of CXCR4 and SDF-1 imaging from the same fields of vision. **(A) **SK-N-SH cells. **(B) **SH-SY5Y cells. Result is a representative image taken from ten separate fields of vision.

Autocrine downregulation of CXCR4 by endogenously derived SDF-1 in the extracellular medium has previously been suggested to explain the low levels of CXCR4 seen on some neuroblastoma cell lines [18]. In an effort to test this theory and more accurately assess basal levels of CXCR4 surface expression an attempt was made to block release of any endogenous SDF-1 and prevent receptor down-regulation by treating cells with Brefeldin A, an inhibitor of vesicular protein secretion. As shown in fig. 4, Brefeldin A treatment did not result in a recovery of CXCR4 on the surface of the low expressing cell line SH-SY5Y, this observation was true for the other low expressing cell lines as well (data not shown). In addition, NGP and SK-N-SH, moderate and high surface expressing cell lines respectively, both showed significant decreases in surface levels as a consequence of Brefeldin A treatment (paired t test, P < 0.005); this observation was also made for the other moderate and high expressing cell lines from our panel (data not shown). In light of the absence of any upregulation of CXCR4 on the surface of Brefeldin A treated cells we decided to determine the presence and abundance of any endogenously derived SDF-1 in the extracellular environment of neuroblastoma cell lines used by us. Quantitative ELISA was performed on conditioned medium collected from cells at 100% confluency. As shown in fig. 5, SDF-1 levels observed in representatives from the high (SK-N-SH or LAN-5), or the low (SH-SY5Y) surface expressing classes were either at, or below background levels of the chemokine measured in culture media (8 pg/ml), and found not to be significant (P > 0.05, One-way ANOVA). The concentration of extracellular SDF-1 for the moderate surface expressing cell line NGP, calculated at 184 pg/ml, was significantly higher than background levels of SDF-1 (P > 0.005, One-way ANOVA); however this level was determined to be below levels used to activate CXCR4 and deemed physiologically irrelevant [18]. With the exception of NGP, SDF-1 levels were observed to be below background levels for all the other cell lines examined regardless of expression class (data not shown). In an attempt to clarify the status of endogenously expressed SDF-1 in neuroblastoma cells localization of the chemokine was assessed using indirect fluorescent immunostaining. SDF-1 was present in both the high surface expressing cell line (SK-N-SH) and its low surface expressing sub-clone (SH-SY5Y) (Figs. 6A and 6B), and appeared to localize predominately at the inner periphery of the plasma membrane. Staining in SK-N-SH cells appearing more focal and polarized with respect to CXCR4, while SH-SY5Y cells showed a more diffuse staining pattern; there was little to no co-localization of SDF-1 and CXCR4 in either cell line. The staining pattern for SK-N-SH was observed for all medium and high CXCR4 surface expressing cell lines in our panel while the pattern for SH-SY5Y was observed for all the low surface expressing lines (Data not shown). Transcriptional expression of SDF-1 was determined in all cell lines examined to confirm their ability to endogenously synthesis the chemokine (Data not shown).

### Regulation of CXCR4 Surface Expression is associated with Structural Heterogeneity

**Figure 7:**
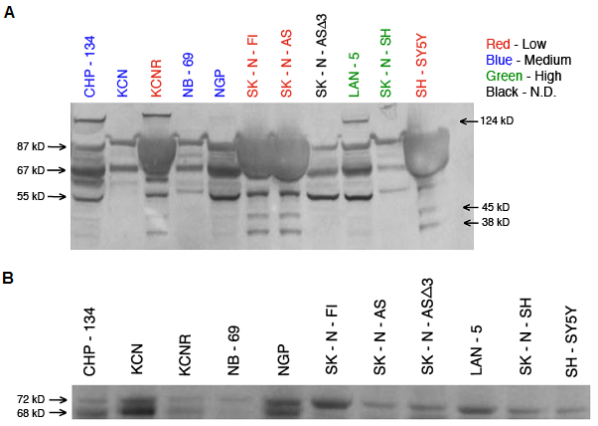
**Analysis of CXCR4 Structural Heterogeneity in Neuroblastoma Cells**. **A) **Western Blot Detection of CXCR4 in Neuroblastoma Cell Lysates. Cell lysates were generated by solubilizing neuroblastoma cells in 1% triton X-100. Detergent soluble proteins were resolved on 4-20% Bis-Tris SDS gels and transferred to nitrocellulose membranes. CXCR4 was detected by probing with a rabbit anti-human CXCR4 polyclonal primary antibody (ab2090), followed by a horse radish peroxidase conjugated donkey anti-rabbit secondary antibody, and then chemiluminescent substrate. CXCR4 was visualized using enhanced chemiluminescent (ECL). The samples represented are as follows: Lane 1- CHP-134, Lane 2-KCN, Lane 3-KCNR, Lane 4-NB-69, Lane 5-NGP, Lane 6-SK-N-FI, Lane 7-SK-N-AS, Lane 8-SK-N-ASΔ3*, Lane 9-LAN-5, Lane 10-SK-N-SH, Lane 11-SH-SY5Y. Cell lines are color coded by surface expression class: Red = Low, Blue = Medium, Green = High, Black = Not Conclusively Determined (N.D.). *SK-N-ASΔ3 is a derivative cell line of SK-N-AS that has been transfected with an expression vector containing the coding sequence for an E3 ubiquitin ligase component Cullin-5 (CUL-5). All samples were loaded equally as determined by comparing actin levels in each lane (Data not shown). **B) **Western Blot Detection of Ubiquitin in CXCR4 Immunoprecipitates. Cell lysates were adsorbed with rabbit anti-human CXCR4 antibodies followed by precipitation with protein G Agarose. Precipitated CXCR4 Immune complexes were resolved on 4-15% Bis-Tris SDS gels and transferred to nitrocellulose membranes. Membranes were probed with a mouse anti-human Ubiquitin monoclonal antibody followed by an HRP-conjugated donkey anti-mouse secondary antibody, and then addition of chemiluminescent substrate. Ubiquitin was visualized by measuring ECL. Sample order is the same as in panel A.

**Figure 8:**
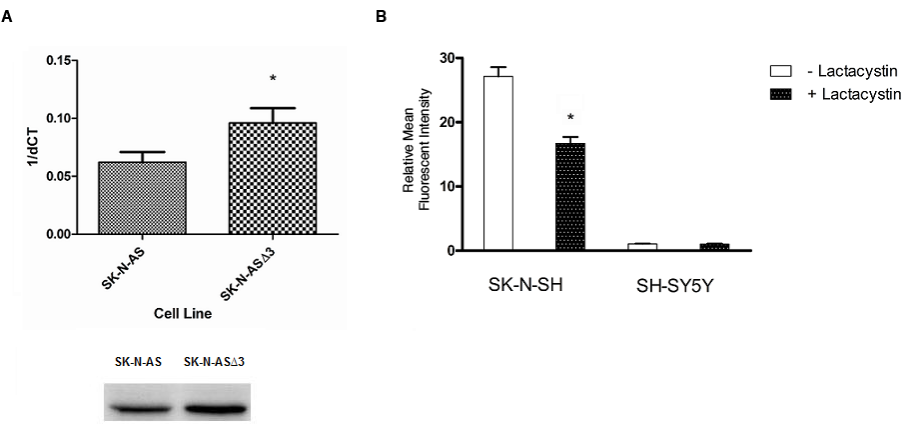
**Evidence for Proteasomal Regulation of CXCR4 Structure-Function. A) **Comparison of Cul-5 Expression in SK-N-AS vs. SK-N-ASΔ3 Cells. Top-Quantitative PCR was performed for 30 cycles of amplification using *CUL-5 *primers and a total of 250 ng of template cDNA from either SK-N-AS or SK-N-ASΔ3. Result represents the mean +/- SD from three experiments (*P < 0.005, paired t-test). dCt values for CUL-5 transcripts were normalized to the endogenous control glyceraldehyde phosphate dehydrogenase. Bottom-Western blot was performed on total cell lysates from SK-N-AS and SK-N-ASΔ3 cells using anti-CUL-5. **B) **Effect of Proteasomal Inhibitor Lactacystin on CXCR4 Surface Expression. Cells were cultured in the presence of 10 μM of lactacystin. After 12 hrs of treatment cells were harvested and processed for FACS analysis. The high CXCR4 surface expressing cell line SK-N-SH and its low surface expressing subclone were both analyzed. PBS was used as a negative control.

CXCR4 has been shown to display significant structural heterogeneity, [22] yet to date no extensive analysis of CXCR4 secondary structure in neuroblastoma has been performed. Western blot analysis of CXCR4 was performed on all the cells in our panel in an attempt to identify any structural heterogeneity of CXCR4 that might further refine our stratification of CXCR4 surface expression. Fig. 7A shows a significant degree of structural heterogeneity for CXCR4 with several isoforms being differentially represented across the three classes of surface expressing lines. Of the various isoforms present five appeared to differentially segregate between the high and low surface expressing classes, and served to help further characterize and distinguish between these two classes. Two major forms of approximately 38 and 45 kilodaltons, corresponded well to reported masses for native and glycosylated forms of CXCR4 respectively [22,23], and were present exclusively in the low CXCR4 surface expressing cell lines. Three major forms of approximately 55, 67, and 87 kilodaltons and equal stoichiometry appeared to primarily associate with the high surface expressing class. It should be noted that to varying degrees these isoforms were also present in the moderate class of surface expressing cell lines, however without the same stoichiometry. The presence of a large diffuse component spanning the 67-87 kDa regions in four of the five low surface expressing cell lines was observed. This material did not appear as a discrete band and was seen on immunoblots with anti-CXCR4 (Fig. 7A), as well as with isotype control immunoblots and on Ponceau S stained membranes prior to immunoblotting (data not shown). CXCR4 has been shown to be ubiquitinated, in a basal and agonist-induced manner, contributing to the degree of heterogeneity frequently observed upon secondary structural analyses [23,24]. To determine if any of the isoforms we observed were ubiquitinated, CXCR4 was immunoprecipitated from cell lysates followed by western blotting with anti-ubiquitin monoclonal antibody. There were two bands of approximately 68 and 72 kilodaltons present to varying degrees in all of the cell lines (Fig. 7B); The 68 kilodalton ubiquitinated molecule appears to correspond to the 67 kilodalton isoform described earlier as one of the three associated with high surface expressing cell lines (Fig. 7A). The 72 kilodalton ubiquitinated molecule is also believed to be a CXCR4 isoform observed in cell lines from both the high and moderate surface expressing lines, albeit not to the same extent. Attachment of ubiquitin serves as a primary signal to identify proteins targeted for turnover by the proteasome. SK-N-AS is known to be deleted at Chromosome 11q23 [25], a region which contains the gene for Cul 5, a component of the E3 ubiquitin ligase complex that mediates proteasomal degradation [26]. Comparison between SK-N-AS and SK-N-AS 3, a derivative cell line transfected with a Cul 5 expression vector, shows a shift occurred from the low surface expressing to high surface expressing CXCR4 structural profile respectively (Fig. 7A); this conversion appears to be associated with changes in Cul 5 expression levels (Fig. 8A). In an effort to further evaluate proteasomal-mediated targeting of CXCR4 as a possible mechanism for regulating its surface expression, neuroblastoma cells were treated with the irreversible proteasome inhibiting agent Lactacystin. Fig. 8B shows treatment of the high CXCR4 surface expressing cell line SK-N-SH with this compound resulted in a significant decrease in surface levels of the receptor relative to untreated cells (P < 0.005, paired t-test). The low surface expressing SH-SY5Y showed no significant changes in CXCR4 levels. These observations were similar for the other high and low surface expressing cells in our panel respectively (Data not shown).

## Discussion

The purpose of this study was to evaluate the expression of CXCR4 in neuroblastoma cells and determine to what extent, if any, expression of this receptor may contribute to the heterogeneity associated with the disease. Using a panel of ten cell lines derived from patients with high-risk neuroblastoma, we compared CXCR4 expression at both the cell surface and transcriptional levels (Figs. 1 &2). Transcriptionally, *CXCR4 *was variably expressed in all of the cell lines examined (Fig. 2A); this observation was made for surface expression as well (Fig. 1B) and subsequently used to stratify cell lines into three categories of expression (Table 1). While overall a direct correlation between transcriptional and surface expression of CXCR4 was observed (Fig. 2C), the margin of difference in transcript levels were not as large as, and hence reflective of, the differences witnessed for surface expression of the protein. It is reasonable to expect that assessment of gene expression using two separate markers (cDNA and cell membrane protein), and hence techniques, might produce results varying in scale based solely on differences in the dynamic range and sensitivity of the assays used. The extent of difference observed when transcriptional and surface expression levels from the same cell line are compared however suggests that some post-transcriptional/translational event is involved in regulating the final expression of CXCR4 on the surface of neuroblastoma cells.

It had previously been suggested that surface expression of CXCR4 on neuroblastoma cells was subject to SDF-1 mediated down-regulation via a negative autocrine loop mechanism [18]. An autocrine mechanism such as the one suggested for CXCR4 regulation should be dependent on the production and subsequent release of SDF-1 from neuroblastoma cells prior to ligand-receptor binding and internalization, and would thus be preceded by the accumulation of ligand in the extracellular environment in a time and cell density dependent manner. While we did observe decreases in CXCR4 surface levels over increasing time and cell confluency (Fig. 3), results from ELISA experiments performed on media from confluent cultures of neuroblastoma cells revealed negligible levels of SDF-1 (Fig. 5), this was despite the detection of SDF-1 transcripts (data not shown) and protein (Fig. 6) inside the cells. It is worth noting there was one cell line, NGP, in which some SDF-1 was detected via ELISA (Fig. 5), however the maximum concentration detected (184 pg/ml) was more than an order of magnitude below the minimum concentration of exogenous SDF-1 (10 ng/ml) used to mediate CXCR4 down-regulation in neuroblastoma cells [18]. This observation, coupled with the failure of the secretory inhibitor Brefeldin A to rescue CXCR4 from down-regulation, strongly suggest a lack of autocrine regulation of CXCR4 in the cell lines we examined. Interestingly, Brefeldin A treatment resulted in a decrease in CXCR4 cell surface expression (Fig. 4), possibly due to disruption of receptor recycling from intracellular vesicles to the cell surface. The presence of detectable SDF-1 at both the mRNA (data not shown) and protein levels (Fig. 6) suggest that this chemokine was produced by the neuroblastoma cell lines we examined. Failure to find SDF-1 in the extracellular media however may be an indication that the cells were either defective in their ability to secrete it, or the appropriate stimulus was not present to elicit its secretion. Although we considered the possibility that neuroblastoma cells in this study were not presented with the appropriate stimuli for secretion of SDF-1, the fact that they were cultured in the presence of 5% fetal bovine serum, a concentration greater than that shown to stimulate SDF-1 secretion from glioblastoma cells [27] strongly suggested otherwise.

We hypothesized that the differential expression of CXCR4 in neuroblastoma cells might be due to post-translational modifications that influenced its trafficking to the cell surface. Structural heterogeneity of CXCR4 due to post-translational modification has been widely demonstrated in a variety of cell types. These modifications have in large part been attributed to glycosylation or ubiquitination of CXCR4 and in some cell types has been associated with different functional responses [22,23]. Given that to our knowledge no such observations have been reported in neuroblastoma cells we examined whether this heterogeneity was present in our cell lines as well, and if so to what extent it might be associated with differential surface expression of the receptor. In our examination of CXCR4 we found the degree of structural heterogeneity to be high (Fig. 7A), observing what appeared to be many of the same isoforms reported by others [22,23]. Of the isoforms we observed we noted some that were more prominently associated with cells in certain surface expression classes such as, the moderate to high class more strongly expressing isoforms of 55, 67, and 87 kDa, and the 38 and 45 kDa isoforms more prominently associated with the low surface expressing class. It is compelling to speculate whether these two classes of isoforms respectively regulate opposing CXCR4 mediated responses such as, the proliferative and cytotoxic signaling in response to SDF-1 stimulation observed in two different neuroblastoma cell lines [17,20]. Another important consideration is the possibility that the association of these structural isoforms with differential expression of CXCR4 at the cell surface is based solely on alterations in epitopes which could result in a loss of recognition by the antibody used for surface staining, however we have seen the same surface expression patterns using a different CXCR4 antibody, MAB-172 (data not shown). Additionally, isoforms similar to the ones observed by us to associate with low surface expression were detected in lymphoblastic leukemic cells via western blotting using the same anti-CXCR4 antibody used in this study to measure surface expression,12G5 [22]. In light of these observations it is unlikely that the differential surface expression witnessed here is reflective of differences in epitope recognition and not protein levels. We also observed differential overexpression of a non-CXCR4 protein component that appeared only in low expressing cell lines (Fig. 7A); this material diffusely localized between 67 and 87 kDa on blots with immune and non-immune antibodies but was not observed however when CXCR4 blots were performed following CXCR immunoprecipitation (data not shown). In our attempts to identify the nature of the various isoforms observed by us we examined our cells for the presence of ubiquitinated CXCR4 via western blotting (Fig. 7B). Two CXCR4 associated proteins were found to cross-react with anti-ubiquitin antibodies, a 68 kDa species which we believe corresponds to the 67 kDa isoform seen in CXCR4 immunoblots from high surface expressing cell lines, and a 72 kDa protein. Based on our observation, and an earlier report suggesting a 68 kDa isoform to represent a non-glycosylated CXCR4 dimer [22], it is likely our 67 kDa isoform represents a ubiquitinated CXCR4 dimer. With regard to the 72 kDa isoform, while we have seen bands of this size on our CXCR4 immunoblots, albeit not as consistently and to a much lesser degree than the other isoforms, it is possible that this particular isoform does not cross react strongly with the monoclonal antibody used in the CXCR4 immunoblot assays; alternatively, it may represent a co-immunoprecipitating molecule that is ubiquitinated. Despite demonstrating the ubiquitination of at least one CXCR4 isoform we were not able to conclusively associate this particular modification with any one class of CXCR4 surface expression. In light of observing posttranslational modification of CXCR4 with ubiquitin in neuroblastoma cells we decided to examine what effect if any, treatment with the irreversible proteasome inhibitor lactacystin would have on surface expression of CXCR4. A previous report had shown an increase in CXCR4 surface expression after lactacystin treatment in monocytes, which was believed to be due to a rescue of the receptor from proteasomal degradation [23]. Our examination revealed a decrease in surface expression of CXCR4 on medium to high expressing neuroblastoma cells following treatment with lactacystin (Fig. 8B); we speculate that this may be due to decreased proteasomal degradation of a negative regulator of CXCR4 surface expression resulting in enhanced downregulation of surface expression. Despite our inability to conclusively demonstrate a direct correlation between ubiquitination patterns and surface expression class we did observe that stable transfection of our SK-N-AS cell line with a CUL-5 expressing construct (Fig. 8A) resulted in a derivative cell line (SK-N-ASΔ3) that was characterized by conversion of its CXCR4 structural profile from one with prominent expression of the 38 and 45 kDa isoforms as observed in the low surface expressing cell lines, to one with strong expression of the 55, 67, 87 kDa isomers observed in high surface expressing cell lines (Fig. 7A). The CUL-5 transcript encodes a component of the E3 ubiquitin ligase which is a part of the proteasomal complex involved in attachment of ubiquitin molecules to targeted proteins and has been associated with breast tumorigenesis [26]; interestingly, this gene is found in a region of chromosome 11q we have shown to be commonly deleted in neuroblastoma and associated with poor outcome [28]. Initial attempts to associate this conversion of CXCR4 isoforms with a change in surface expression level were not successful; however we did notice that this change resulted in SK-N-AS, one of the more aggressively growing and tumorigenic cell lines, becoming much less tumorigenic in nude mice (data not shown). To our knowledge the only E3 ligase associated with CXCR4 is atrophin interacting protein 4 (AIP4) [29]; these observations have in large part been made in cells of hematopoeitic lineage. Given that in the proteasomal complex there is a far greater diversity of E3 components than E1 components, and to a lesser extent E2 components, E3 ligases confer a majority of the specificity for substrate recognition to the proteasomal complex [30]. Based on this, the possibility that in neuroblastoma cells an E3 ligase other than AIP4, possibly Cul-5, mediates modification of CXCR4 is intriguing. The combined observations that down-regulation of CXCR4 occurred in the absence SDF-1 stimulation, and in response to lactacystin treatment, may potentially underscore a novel mechanism of GPCR regulation. Such a mechanism might involve proteasomal mediated modification of CXCR4, possibly with ubiquitin, which targets CXCR4 to the surface of neuroblastoma cells as opposed to lysosomal degradation. If such a mechanism were to exist then the ligand independent downregulation we observed could represent the kinetic turnover of post-translationally modified CXCR4 whereby a molecule such as ubiquitin is cleaved off of CXCR4 resulting in its internalization and degradation. An example of this type of mechanism is exhibited in the constitutive activation of another seven transmembrane domain receptor, Protease-activated receptor-1 (PAR1) [31].

## Conclusions

Taken together, our data show that CXCR4 is widely expressed at variable levels on the surface of patient-derived neuroblastoma cell lines. We believe this variability is in large part due to structural heterogeneity of the receptor as a result of post-translational modifications and that these modifications are in some small part due to ubiquitination, but more probably to a greater extent due to oligomerization. Additionally, it appears that direct or indirect regulation by proteasomal components not yet identified are involved in the regulation of CXCR4 on the surface of neuroblastoma cells, and that this regulation can occur independent of stimulation by agonist as evidenced by the absence of any apparent endogenously derived SDF-1. Furthermore, the apparent lack of endogenous SDF-1 involvement in our studies demonstrate that regulation of CXCR4 surface expression can occur in a non-autocrine, SDF-1 independent manner, which suggests that this chemokine is not the sole obligatory signal for all CXCR4 mediated activity, a likelihood which was first underscored in a study using neuroblastoma cells that were metastatically active and positive for CXCR4 surface expression yet desensitized to SDF-1 [19]. Initial efforts by our laboratory to demonstrate a functional difference between CXCR4 mediated responses in our low versus high surface expressing cell lines were unsuccessful (data not shown) however the contribution of other, not yet identified factors which may be present in the much more ï¿½factor-richï¿½ environment of the biological stroma can not be ruled out. Recent work has demonstrated that expression of CXCR4 in neuroblastoma cells can be differentially regulated by exposure to stromal components, and that the nature of this regulation can vary dependent on both the cell line being examined and the tissue origin of the stroma. In their study the authors identified liver stromal-derived IFN-γ and IL-5γ as two components having positive and negative regulatory effects respectively on CXCR4 expression in one of their cell lines and were able to directly correlate expression with cell migration. Although not reported it would be interesting to know if the authors observed any of the structural heterogeneity we identified to be associated with the differential expression and chemokine/receptor modulations witnessed in their study. It should be noted that the authors used mouse-derived stromal cells to influence CXCR4 expression on human neuroblastoma cells, however in the context of our findings the possible contributory effects from other stromal-derived cytokines such as IFN-γ and IL-5γ on the expression and function of CXCR4 in our system warrant investigation. Further work will focus on identifying the nature of the CXCR4 modifications that exist in neuroblastoma cells and the extent, if any, to which these isoforms regulate the various CXCR4 mediated responses.

## Competing interests

The authors declare that they have no competing interests.

## Authors' contributions

AJC conceived of and initiated this project, as well as performed indirect immunoflourescence and western blot assays, data analysis, writing and editing of manuscript. CL performed cell culture, FACS and ELISA assays. RC performed cell culture, FACS and RT-PCR assays. JMM performed data analysis and manuscript editing, and also provided critical expertise on the biology of neuroblastoma cells. All authors have read and approved the final manuscript.
